# Intrathecal Pseudodelivery of Drugs in the Therapy of Neurodegenerative Diseases: Rationale, Basis and Potential Applications

**DOI:** 10.3390/pharmaceutics15030768

**Published:** 2023-02-25

**Authors:** Menéndez-González Manuel, Bogdan-Ionel Tamba, Maxime Leclere, Mostafa Mabrouk, Thomas-Gabriel Schreiner, Romeo Ciobanu, Tomás-Zapico Cristina

**Affiliations:** 1 Facultad de Medicina y Ciencias de la Salud, Universidad de Oviedo, Calle Julián Clavería s/n, 33006 Oviedo, Spain; 2 Department of Neurology, Hospital Universitario Central de Asturias, Avenida Roma s/n, 33011 Oviedo, Spain; 3 Instituto de Investigación Sanitaria del Principado de Asturias, Avenida Roma s/n, 33011 Oviedo, Spain; 4 Advanced Research and Development Center for Experimental Medicine (CEMEX), “Grigore T. Popa” University of Medicine and Pharmacy, Universitatii Str., No. 16, 700155 Iasi, Romania; 5 Adaptative Supramolecular Nanosystems Group, Institut Europeen des Membranes, University of Montpellier, ENSCM-CNRS, Place E. Bataillon CC047, 34095 Montpellier, France; 6 Refractories, Ceramics and Building Materials Department, National Research Centre, 33 El Bohouth St. (Former EL Tahrir St.), Dokki, Giza 12622, Egypt; 7 Faculty of Medicine, University of Medicine and Pharmacy “Carol Davila”, 050474 Bucharest, Romania; 8 Department of Neurology, University of Medicine and Pharmacy “Gr. T. Popa”, 700115 Iasi, Romania; 9 Department of Electrical Measurements and Materials, Faculty of Electrical Engineering and Information Technology, Gheorghe Asachi Technical University of Iasi, 700050 Iasi, Romania

**Keywords:** drug delivery systems, intrathecal pseudodelivery, neurodegenerative diseases, intrathecal device, nanoporous membranes

## Abstract

Intrathecal pseudodelivery of drugs is a novel route to administer medications to treat neurodegenerative diseases based on the CSF-sink therapeutic strategy by means of implantable devices. While the development of this therapy is still in the preclinical stage, it offers promising advantages over traditional routes of drug delivery. In this paper, we describe the rationale of this system and provide a technical report on the mechanism of action, that relies on the use of nanoporous membranes enabling selective molecular permeability. On one side, the membranes do not permit the crossing of certain drugs; whereas, on the other side, they permit the crossing of target molecules present in the CSF. Target molecules, by binding drugs inside the system, are retained or cleaved and subsequently eliminated from the central nervous system. Finally, we provide a list of potential indications, the respective molecular targets, and the proposed therapeutic agents.

## 1. The BBB, the CSF, and the Neurodegenerative Diseases

**Figure 1:**
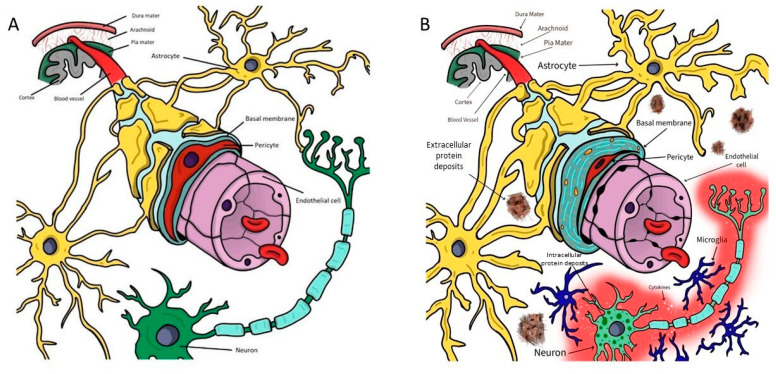
(**A**)**.** The complex structure of the neurovascular unit in physiological conditions. All components interact anatomically and chemically in a complex web to maintain its functions. Endothelial cells (purple), which make up the main part of the BBB, are characterized by high selectivity in transcellular transport, due to the tight junctions that fuse them together and restrict diffusion across the blood vessels. Pericytes (red) are essential cells in maintaining the structural and functional properties of the BBB and share a common basement membrane (blue) with endothelial cells. Astrocytes (yellow) are involved in supportive processes and have a strategic localization between neurons (green) and other components of the BBB, with their specialized end feet extending to the walls of the blood vessels. (**B**). The most relevant pathophysiological changes of the neurovascular unit in NDD. Many of the homeostatic processes of the BBB are impaired in NDD. Vascular integrity is impaired by damage to the endothelial cells (purple), which lose their impermeability in the tight junctions, along with atrophy of pericytes (red), astrocyte endfeet swelling (yellow), and collagen and laminin accumulation in the basal membrane (blue). Aggregates of protein build up and organize in plaques that surround the astrocytes and neurons. This causes neuroinflammation with the secretion of inflammatory cells and cytokines, with the central role played by microglia (dark blue). Within neurons (green), proteins may also accumulate in intracellular aggregates, which are associated with the accumulation of glial cells and neuronal dysfunction. Modified from Schreiner et al. [7] (Magda Pîrțac originally designed this figure by using Adobe Fresco).

Neurodegenerative diseases (NDD) are a group of disorders of the central nervous system (CNS) that cause progressive death of nerve cells and loss of function in the brain and spinal cord. The CNS compartments are represented by the parenchyma of the brain and spinal cord, including the intracellular space with the intracellular fluids (ICF) and the extracellular space with the interstitial fluid (ISF), and the cerebrospinal fluid (CSF) space. The tissues of the CNS are separated from the systemic circulation by the blood-brain barrier (BBB) and blood-CSF barrier (BCSFB) [1,2]. These barriers protect the CNS from endogenous and exogenous compounds present in the systemic circulation and are essential to ensure the proper function of the CNS. The BBB is a complex and highly selective structure, a veritable border for numerous substances (including macromolecules) found in the systemic circulation in their passage to the CNS [1]. This protective role is mainly the consequence of the presence of an endothelial layer with special features, with flattened, polarized endothelial cells that have an increased mitochondrial content, minimal pinocytic activity, a lack of fenestrations, and are closely linked via an elaborate protein network consisting of tight junctions and adherens junctions [2] (Figure 1A). Besides the almost non-existent paracellular passage of molecules, the presence of different highly selective transporters on endothelial cells limits the free entry of drugs to the CNS [3]. Despite acting as a carrier by allowing the penetrance of glucose, vitamins, lipid-soluble molecules, and gases (carbon dioxide and oxygen) from the blood toward the CNS, the BBB serves as a shield against neurotoxins, but also against potentially therapeutic substances. Additionally, these physical-chemical properties of the BBB are maintained in physiological conditions thanks to the intercellular crosstalk between endothelial cells and the other components of the BBB (pericytes, astrocytes, neurons). Regarding the BCSFB, it is located in the choroid plexus of the brain ventricles, and it is composed of a cuboidal cell epithelium with adhering Kolmer cells, a highly vascularized stroma with connective tissue, and the brain capillary endothelium [4]. According to the classical paradigm, the primary role of the choroid plexus epithelial cells is the secretion of CSF into the brain ventricles; however, recent research acknowledges the protective role of the BCSFB for the cerebral parenchyma [5]. In contrast to the BCSFB and BBB, the CSF and the ISF are not tightly separated. Even large molecules up to the size of albumin can move passively from/to the ISF and the CSF. Circulation of CSF and ISF around and through the CNS transports not only fluids but also any solutes they carry, including nutrients, drugs, and metabolic wastes. Impairment of this circulation, which is more intense during sleep, has profound implications for NDD [6]. 

**Figure 2:**
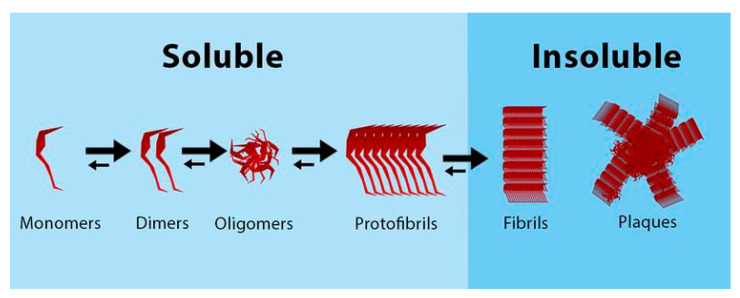
Aggregation of proteins from monomers to plaques. Soluble monomers aggregate together to form dimers and oligomers, that can form soluble protofibrils. These protofibrils aggregate to form insoluble fibrils, that can form plaques. The process is dynamic and bidirectional, therefore complex aggregates can disaggregate into less complex aggregates. Modified from Kok et al., 2022 [8].

Pathologically, besides cellular loss, most NDD exhibit molecular hallmarks such as beta-amyloid (Aβ), tau, α-synuclein, mSOD1, and TDP-43. Disease-distinctive proteins exist in different states that aggregate between them: soluble monomers aggregate together to form dimers and oligomers, that can form soluble protofibrils. In turn, protofibrils aggregate to form insoluble fibrils, that eventually deposit in the form of plaques or tangles [8]. Soluble proteins are present in the CSF, and in equilibrium—either direct or inverse, depending on the molecule and the stage of the disease—with the concentration in the ISF (Figure 1). Similarly, polyglutamine (polyQ) diseases are a group of genetic NDD caused by the abnormal expansion of a CAG trinucleotide repeat that is translated into an expanded polyQ sequence in the disease-causative proteins. The expanded polyQ sequence itself plays a critical disease-causative role in the pathogenic mechanisms underlying these diseases. The most common pathogenic mechanism in polyQ diseases is related to the fact that the expanded polyQ sequence promotes a conformational transition from the native monomer into the β-sheet-rich monomer, followed by the formation of soluble oligomers and finally insoluble aggregates with amyloid fibrillar structures (Figure 2). The intermediate soluble species including the β-sheet-rich monomer and oligomers exhibit substantial neurotoxicity [9].

From this perspective, the central event in the pathophysiology of NDD is a proteostasis imbalance leading to protein aggregation overwhelming the proteostasis capacity of brain cells (e.g., autophagy-lysosome and ubiquitin-proteasome systems), and interfering with the ability of neurons to cope with pathogenic proteins [10,11], which accumulate and deposit intracellularly and/or extracellularly (Figure 1B). Eventually, protein aggregates lead to neuronal cell death, frequently mediated by activated tyrosine kinases. Not in vain, the exquisitely tuned activity of protein kinases is essential to maintaining cellular homeostasis [12]. Whereas loss-of-function variants are generally associated with cancer, gain-of-function variants are associated with NDD. Since these pathways are crucial for degrading aggregate-prone proteins and dysfunctional organelles such as mitochondria, they help maintain cellular homeostasis. As post-mitotic neurons cannot dilute unwanted protein and organelle accumulation by cell division, the nervous system is particularly dependent on autophagic pathways. This dependence may be a vulnerability as people age and these processes become less effective in the brain. The origin of proteostasis imbalance may be due to a genetic origin and/or acquired causes. Today, the pathogenic mechanisms underlying most genetic NDD are generally known, yet we do not have a clear understanding of the etiologies of sporadic NDDs. In sporadic NDD, some risk or protective factors have been identified (genetic polymorphisms, style of life including exercise, sleep, and diet), but the precise links between these factors and the pathogenic mechanisms leading to proteostasis imbalance are yet to be deciphered. In any case, the formation of aggregates of these proteins may be the consequence of different pathogenesis, including a variable combination of increased synthesis, synthesis of structurally abnormal forms, and decreased degradation, either by intracellular (autophagy, microglia) or extracellular systems [13,14,15,16]. A decrease in their clearance to compartments outside the brain parenchyma has been identified as a relevant contribution to protein accumulation in the CNS fluidic systems, due to the impairment of the BBB, the CSF flow, and the glymphatic system [17,18,19,20,21,22,23]. Protein degradation involves enzymes contributing to clear target molecules, such as neprilysin or insulysin, that clear Aβ [24]. The soluble fraction of the triggering receptor expressed on myeloid cells 2 (sTREM2) is a bioactive molecule capable of binding ligands, activating microglia, and regulating immune responses during neurodegenerative processes. While sTREM2 promotes microglial survival and stimulates the production of inflammatory cytokines, variants of sTREM2 are less potent in both suppressing apoptosis and triggering inflammatory responses. In Alzheimer’s disease (AD), wild-type sTREM2 binds oligomeric Aβ and acts as an extracellular chaperone, blocking and reversing Aβ oligomerization and fibrillization, and preventing Aβ-induced neuronal loss in vitro. Levels of sTREM2 in CSF fall prior to AD clinical onset, rise in early AD, and fall again in late AD. Subjects with higher sTREM2 levels in CSF progress more slowly into and through AD than do subjects with lower sTREM2 levels, suggesting that sTREM2 may protect against AD [25,26,27,28]. 

In parallel to proteostasis disbalance, a common feature in NDD is chronic immune activation, in particular of astrocytes and microglia, the resident macrophages of the central nervous system [29]. The CSF immune system dysregulates during healthy brain aging and especially during neurodegenerative processes. Monocytes upregulate lipid processing genes with age in cognitively normal CSF, and particularly in neurodegeneration [30]. The release of aggregated pathogenic proteins such as Aβ, tau, α-synuclein, mSOD1, and TDP-43 into the extracellular space, drives the changes of microglia and astrocytes into their pro-inflammatory phenotypes (Figure 1B). The pro-inflammatory-phenotype astrocytes and activated microglia release pro-inflammatory factors, such as interleukins and tumor necrosis factor α (TNF-α), which act as mediators dysregulating the synaptic function, the BBB, the metabolic function, and CSF and blood flow [5,29,30,31,32,33,34]. Altogether, the predominance of the pro-inflammatory state results in the increase of pro-inflammatory factors and in a decrease in the protein clearance; and ultimately in disease progression.

## 2. The Problem of Drug Delivery to the CNS and Its Many Explored Solutions

The administration of drugs targeting the CNS poses challenges. The main difficulty is related to poor drug penetration through the BBB, as multiple in vivo, in vitro, and in situ experiments have demonstrated [35]. Small molecule diffusion through BBB operates similarly to solute-free diffusion through biological membranes. The probability of a given small molecule passing through the BBB can be predicted based on its molecular weight (MW) and structure. If the MW is greater than 450 Daltons, and/or the drug’s structure includes polar functional groups that form more than seven hydrogen bonds, then its transport through the BBB will be low unless there is carrier-mediated transport [36]. Conversely, if the MW is less than 450 Daltons and the drug forms seven or fewer hydrogen bonds with water, then its transport through the BBB may be significant, provided that it is not a substrate for an active efflux transporter. In certain pathological conditions such as stroke or cancer, the BBB experiences structural and functional changes that damage the central nervous system. BBB leakage enables increased immune cell traffic and substance passage to the interstitial fluid. The BBB can be opened through physical interventions like hyperosmotic infusions or focused ultrasounds, which facilitate the entry of drugs such as mAbs [37,38]. When it comes NDD, BBB dysfunction is linked to chronic inflammation, heightened oxidative stress, and the pathological accumulation of misfolded proteins. These factors impede drug delivery to CNS in comparison to acute conditions. Furthermore, the BCSFB may also be altered in NDD, undergoing similar cellular and molecular changes to BBB alterations, leading to permeability changes [4,36,37,38].

**Figure 3:**
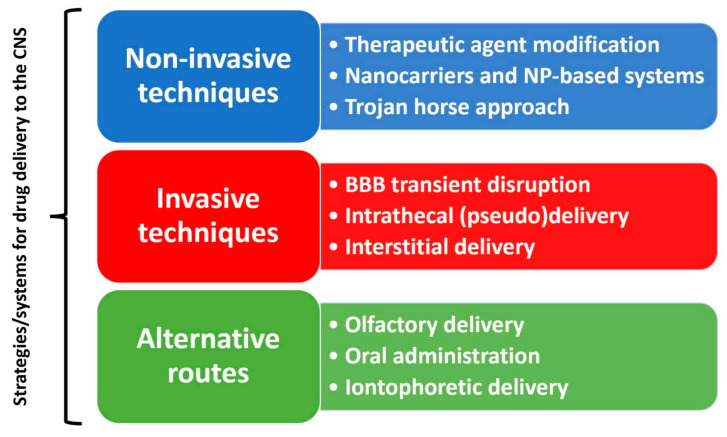
Current strategies/systems for drug delivery to the CNS. (Color code: blue—non-invasive methods, red—invasive methods, green—alternative methods; Abbreviations: BBB—blood-brain barrier; CNS—central nervous system; NP—nanoparticle).

In recent years, a multitude of brain drug delivery technologies have emerged, including trans-cranial delivery, CSF delivery, BBB disruption, lipid carriers, prodrugs, stem cells, exosomes, nanoparticles, gene therapy, endogenous BBB carrier-mediated transport and receptor-mediated transport systems [36]. As presented in Figure 3, there are available at present both invasive and non-invasive techniques, while alternative routes shunting the natural brain barriers are also being explored. Nanoparticle (NP)-based systems have shown promising potential as precision medicines that can effectively penetrate the BBB by crossing, avoiding, or disrupting the BBB. Diverse systems, including liposomes, micelles, polymeric NPs, solid-lipid NPs, and inorganic NPs, have been investigated for NP drug loading to treat NDD [39,40]. Exosomes are extracellular vesicles secreted by a wide variety of cells, and their primary functions include intercellular communication, immune responses, human reproduction, and synaptic plasticity. Due to their natural origin and molecular similarities with most cell types, exosomes have emerged as promising therapeutic tools for numerous diseases, particularly NDD [41].

Invasive techniques rely on implantable devices accessing the CSF-intrathecal (IT), intraventricular (IVT) or ISF intraparenchymal delivery. In contrast to the extensive use of the CSF for diagnostic purposes, the CSF has not been frequently regarded as a target biological fluid for therapies for CNS conditions because it requires invasive procedures and systems. Today, few drugs are delivered in the CSF, mainly because it is an invasive procedure not without risks. However, in recent decades, therapies addressed at the CSF have gained some momentum as a result of advanced treatments such as gene therapies and replacement enzymatic therapies which need IT or IVT delivery. While the IT drug delivery pathway is still regarded as an experimental approach in neurodegenerative diseases, this route has long been successfully used in the treatment of other pathological conditions and in the symptomatic therapy of pain and spasticity [42,43,44,45]. More recently, intrathecal infusion of Nusinersen, an antisense oligodeoxynucleotide (ASO), was approved by FDA and EMA for the treatment of spinal muscular atrophy (SMA) [46]. Several ASOs have been tested in clinical trials for their ability to treat brain or spinal cord parenchyma by injecting drugs into the lumbar CSF. One such ASO is Tominersen, which targets the huntingtin mRNA of Huntington’s disease (HD). Another ASO, Tofersen, targets the superoxide dismutase 1 (SOD1) mRNA in SOD1-dependent amyotrophic lateral sclerosis (ALS). [47].

Controlled drug delivery systems (DDS) seek to improve patient compliance by increasing therapeutic efficacy, extending drug release time and stability, increasing drug bioavailability, reducing side effects, and reducing dosage frequency. Moreover, DDS contribute to the safety of pharmaceuticals during their whole delivery period by serving as various kinds of protective barriers that enclose them, minimizing the loss of active ingredients and any harmful impacts on patients. They are typically constructed at nanometric and micrometric levels in order to combine several qualities such as site-specificity, endurance, or external stimuli sensitivity [48,49,50].

Intrathecal pumps are a good example of implantable DDS targeting the CNS. Intrathecal pumps consist of an electromechanical pump cased with a metal reservoir that stores the medication, and a catheter that is implanted in the spinal intrathecal space to deliver the medication from the pump to the CSF, thus accessing the CNS where the medication takes effect. Two types of pumps are available: a constant rate pump delivers the medication at a constant rate, and a programmable pump delivers the medication according to a rate determined by a computer program [51,52]. While intrathecal pumps offer good control of the rate of drug release, and enable effective low dosing, thus reducing the incidence and severity of drug-derived adverse effects, they are not exempt from complications, including the risk of overdose as a result of incorrect pump programming, pump failure, CSF leak, granuloma formation, obstruction of CSF flow, and infections [53,54].

## 3. Clearing the CSF as a Therapeutic Strategy in Neurodegenerative Diseases

**Figure 4:**
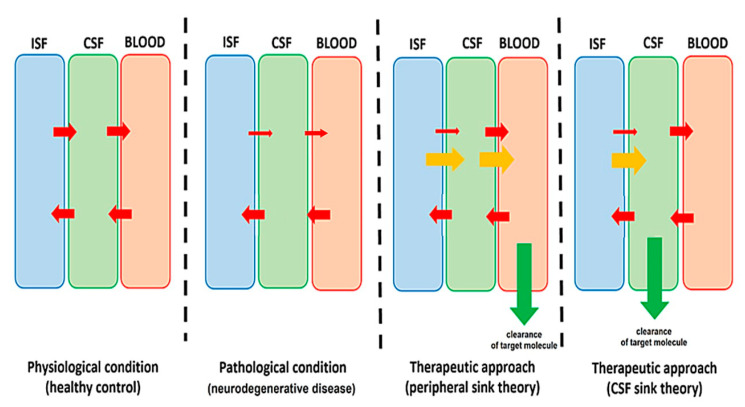
Representation of target molecule dynamics in four scenarios. From left to right: physiological condition (healthy subject), pathological condition (untreated neurodegenerative disease), and pathological condition treated with peripheral sink therapeutic approach or pathological treated with CSF-sink therapeutic approach. Red arrows represent spontaneous equilibrium of target molecules between CNS fluid compartments, green arrows represent pathways of therapeutic clearance of target molecules and orange arrows represent the secondary equilibrium of target molecules between CNS fluid compartments after therapy. Modified from Schreiner et al [60].

Different approaches have been investigated with the aim of removing pathogenic proteins from the CNS, including inhibition of protein synthesis, and promoting protein degradation. Most therapeutic strategies addressed to enhance the clearance of brain proteins rely on clearing them from the periphery [55]. Disease-modifying therapies, such as monoclonal antibodies (mAb) against target molecules, recently started showing clinical benefits in some NDD at least [56]. However, safety remains a concern, since peripherally administered mAbs may lead to serious side effects, such as immunologically mediated amyloid-related imaging abnormalities (ARIA) after anti-Aβ mAb therapies [57]. However, there might be a much more direct way of clearing proteins from the brain than removing them from the plasma: removing them from the CSF. This is the rationale of the so-called “CSF-sink therapeutic hypothesis” [58,59,60] (Figure 4).

Indeed, several attempts have been made to treat neurodegenerative diseases using different CSF filtration systems. In ALS, extracorporeal CSF filtration was shown to successfully mitigate the neurotoxic capacity of CSF from subjects with sporadic Amyotrophic Lateral Sclerosis (ALS) in vitro [61] and in a mouse model [62]. However, a very small randomized, controlled, and open study in the nineties, concluded that filtration of 200–250 mL CSF daily, over five days, did not seem to have a substantial therapeutic effect in patients with ALS [63]. In a single case of familial ALS, there was subjective, but no objective, improvement of the patient immediately after CSF filtration and two weeks later [64].

It is worth revising methods aimed at CSF dilution or enhancing CSF flow, as they are closely related to CSF-sink therapeutic strategy. In AD, mechanical dilution of CSF has long been a proposed therapeutic approach [65]. CSF shunts such as ventriculo-peritoneal, ventriculo-pericardial, ventriculo-atrial and lumbo-peritoneal shunts are the recommended therapy for communicating hydrocephalus. Noteworthy, shunting procedures delay intracerebral deposition of Aβ in patients with communicating hydrocephalus [66]. COGNIShunt is a system for a continuous, low-flow ventriculoperitoneal shunt (Eunoe, acquired by Integra Lifesciences). Results of the clinical trial showed that the difference between treatment groups, while still favoring the COGNIShunt group, was not statistically significant [67]. Arethusta (Leucadia Therapeutics) is a system based on an implantable device to restore CSF flow across the cribriform plate, with no clinical reports yet.

## 4. Intrathecal Pseudodelivery of Drugs: Concept, Advantages, and Disadvantages

Therapeutics such as enzymes, antibodies, and even transport proteins (e.g., albumin), which are mostly intended to link with molecular targets to be removed from the organism, do not really need to be released in the fluid or tissue to action. In fact, binding to the molecular target can be achieved regardless of the compartment. With this in mind, IT pseudodelivery of drugs is a novel concept to administer drugs to treat CNS conditions relying on the CSF-sink therapeutic strategy [60], by means of implantable DDS to put in touch therapeutics with molecular targets inside of the device, without delivering to the biological fluid (hence the name “pseudo”-delivery). The key component in the device is a smart design of customized nanoporous membranes that allow the influx of small molecules (targets) at the time of preventing the efflux of therapeutics of larger molecular size (nanosieve) see the Supplementary Materials for a short animation.

Functional nanoporous materials are an important class of nanostructured materials because of their tunable porosity and pore geometry (size, shape, and distribution) and their unique chemical and physical properties. Progress in developing a broad spectrum of nanoporous materials has accelerated their use for extensive applications in biomedical fields [68]. Nanoporous membranes are natural or synthetic membranes that can be made from a variety of materials and can be fabricated in different configurations including pore size, surface coating, geometry, and pore distribution, providing unique mass transport characteristics that have numerous potential biological and medical applications that involve isolating, sorting, sensing, and releasing biological molecules. Nanoporous membranes are of great interest in drug delivery because they offer a secure delivery system for medications and stop bodily enzymes from breaking them down and because they can be tailored-made and fine-tuned for precise control of the rate of drug delivery or to exquisitely adjust the selective molecular permeability [69,70]. While a few years ago there were technical challenges for the successful application of nanoporous membranes to controlled drug delivery applications—including the need for biocompatibility, the reduction of risk of infections, and the reduction of risk of biofouling [71] most of these challenges have already been overcome and solutions are now being optimized [72,73]. Nanoporous membranes can be used as stand-alone DDS or assembled into complex DDS.

IT pseudodelivery is the first DDS to be endowed with nanoporous membranes acting on the CNS [72,74,75]. Devices for IT pseudodelivery of drugs look similar to intrathecal pumps as they also have a subcutaneous reservoir and an intrathecal catheter accessing the CSF. However, they are not necessarily endowed with electromechanical pumps. The mechanism of action depends on the use of nanoporous membranes enabling selective molecular permeability [72,75]. On one side, the membranes do not allow crossing of drugs, but on the other side, they allow crossing of the target molecules present in the CSF. Target molecules bind drugs inside the system, thus being trapped or cleaved and eliminated from the CNS (a short simulation illustrating the mechanism of action can be found as a Supplementary Materials). Drugs are not released from the reservoir to the organism, and they can be replaced as needed percutaneously through self-sealing septa in the reservoir.

Not every target molecule or drug is suitable to be targeted/used via pseudodelivery. For a disease to be suitable to be treated using IT pseudodelivery, three conditions must be met:

1. A target molecule should be present in the CSF (soluble). This should be identified as potentially “toxic” or “pathogenic” and involved directly (aggregating proteins) or indirectly (mediators) in the physiopathology of the disease.
2. A drug acting specifically on the target molecule is needed. This can be an antibody, an aptamer, an enzyme, or any other compound that has specificity over the target molecule and either binds or cleaves the target molecule.
3. A significant size difference should exist between the target and drug molecules. While other physicochemical features may also play a role (such as electrostatic charge), the size difference is the main feature driving the selective molecular permeability through nanoporous membranes.

While the development of this therapy is still in the preclinical stage, it offers promising advantages over traditional routes of delivery. Being target-selective provides advantages over other CSF clearance systems since the level of other proteins —not involved in disease pathogenesis—would be preserved. It also provides important advantages over “standard” peripherally administered drugs, including the following: 1. Acting continuously, on the CSF directly, is expected to be much more effective than acting peripherally. 2. Immunoisolation of drugs impedes immune responses, fully avoiding immunologically mediated side effects reported with biological drugs systemically administered [74,75].

In contrast, potential adverse effects related to the intrathecal system implantation and functioning should be taken into consideration, with expected local complications similar to those seen with intrathecal pumps, such as CSF leak, hemorrhage, and infection, along with device-derived problems such as CSF flow obstruction or even device disconnection [74,75].

## 5. Potential Applications of Intrathecal Pseudodelivery of Drugs: Diseases, Targets, and Relevant Drugs

The field of disease-modifying therapies for NDD is one of the hottest topics in medicine nowadays. Despite a myriad of studies, no effective disease-modifying treatment is available at the present for most of these conditions [76] while the first disease-modifying therapies for AD have been recently approved with some controversy regarding their efficacy and safety [77,78]. However, much knowledge has been accumulated regarding the molecules and cellular pathways involved in the pathogenesis of NDD that can become valuable targets for future therapies. Different classes of therapeutics are suitable to be used via intrathecal pseudodelivery in the treatment of NDD. Table 1 summarizes the most relevant NDD, their known molecular targets, and the therapeutic agents proposed to be applied through this route, based on previous evidence on the drugs’ mechanism of action. There is little research testing IT pseudodelivery in these conditions yet, hence this list should be considered just as therapeutic hypotheses today.

Monoclonal antibodies (mAbs) directed against misfolded proteins such as Aβ, Tau protein, or α-synuclein are a first choice when considering IT pseudodelivery, as they have been demonstrated to be effective when administered intravenously in many studies [118]. Moreover, mAbs targeting Aβ were approved very recently for the treatment of AD in humans (see Aducanumab [79] and Lecanemab [77]). mAbs is the only class of therapeutics with in vivo studies published via intrathecal pseudodelivery, which showed feasibility, good safety, and histological efficacy in animal models of AD [74,75]. Aptamers are an interesting class of compound that could replace antibodies in the near future, as they can also be used for therapeutic purposes within the pseudodelivery device. Compared to currently available mAbs, aptamers have some advantages such as a smaller size and mass, lower immunogenicity, greater replicability, and a greater level of control (high durability, sensitivity, and specificity) [80]. Similarly, antibodies and aptamers binding other pathogenic proteins such as Alpha-syn, Tau, TDP43, or mutant HTT might be of interest to treat other NDD via the pseudodelivery route, even if they failed when systemically administered for safety or efficacy reasons [58,85,86,96,97,103,105,115].

Other molecules binding pathogenic proteins can be of interest. For instance, human serum albumin (HSA) is a natural buffer of Aβ. A promising approach to AD prevention is to reduce the concentration of free Aβ by targeted stimulation of the interaction between HSA and Aβ. This approach can be implemented by pseudodelivering albumin alone [81] or in combination with agents increasing the affinity of HSA to Aβ through the action of HSA ligands [82].

Another therapeutic possibility is to act on the enzymatic dysfunction, a relevant example being the switch from the non-amyloidogenic pathway to the amyloidogenic one in AD [119]. In the same manner, compensating for the malfunctioning enzymes or even using different enzymes (from the family of membrane metallo-endopeptidases such as neprilysin and other Aβ cleaving enzymes [24]) inside the pseudodelivery device can be a smart option considering the high CSF throughput. 

Protein conformation stabilization and aggregation inhibition that targets the upstream of the insoluble aggregate formation would be a promising approach toward the development of disease-modifying therapies for most NDD, particularly for polyQ diseases. PolyQ aggregation inhibitors of different chemical categories, such as intrabodies, peptides, and small chemical compounds, have been identified through intensive screening methods [116,117]. Among them, those with high molecular sizes are suitable to be used via IT pseudodelivery. The same approach could be used to inhibit the aggregation of Aβ, Tau, alpha-synuclein, SOD, and TDP43 [83,87,99,110,111,113,120]. In addition, clearing cofactors promoting protein aggregation, such as iron or tyrosine kinase, are an alternative way of inhibiting protein aggregation [121]. Interestingly, some nanomaterials such as polyoxometalates may also work as inhibitors of amyloid aggregation [84] and might be suitable to be used as therapeutic agents through this route.

Finally, another clear target in NDD are molecules involved in inflammation such as anti-TNF-α. According to several reports, anti-TNF-α agents may affect amyloidosis in inflammatory/autoimmune diseases, such as rheumatoid arthritis and familial Mediterranean fever [121]. Indeed, perispinal administration of the anti-TNF-α medication etanercept (a fusion protein produced by recombinant DNA) has been reported effective in cognitive improvement in one single case report [92], and similar results were obtained in animal studies [32]. Comparable results were noticed for infliximab, a chimeric monoclonal antibody already approved for the treatment of multiple autoimmune diseases such as Crohn’s disease, rheumatoid arthritis, and psoriasis. A study indicated that intracerebroventricular administration of infliximab reduced Aβ plaques and tau phosphorylation in APP/PS1 mice [93] and resulted in cognitive improvement in a human case [94], while recent research confirms the protective cerebral effects (reduced microgliosis, neuronal loss, and tau phosphorylation) of TNF-α inhibitors in a transgenic mouse model of tauopathy [114]. These results are encouraging, indicating that IT infliximab offers an alternative therapeutic approach for AD, and potentially for other neurodegenerative disorders whose pathogenesis involves TNF-α such as PD [101] and ALS [113]. Clinical trials for different conditions have shown a detrimental effect of TNF-α antagonists in advanced heart failure and anti-TNFs are associated with an increased risk of infection. Rare case reports of drug-induced lupus, seizure disorder, pancytopenia, and demyelinating diseases have been noted after systemic treatment with TNF-α antagonists [122,123]. Meanwhile, chronic dosing with a brain-penetrant biologic TNF-inhibitor induced hematology and iron dysregulation in aged APP/PS1 mice [95]. In this regard, IT pseudodelivery of anti-TNF-α agents may offer a safer route of administration.

Drugs targeting the complement component C5, CD19 on B cells, and the inter-leukin-6 (IL-6) receptor, have been used for the treatment of patients with refractory inflammatory CNS diseases. Particularly, Tocilizumab, a humanized, monoclonal antibody against the IL-6 receptor, has been tested for neurologic indications, such as neuromyelitis optica [124] or primary CNS vasculitis [125]. Tocilizumab has also been tested in ALS [112] and proposed in PD [100] and AD [91] As IL-6 is present in the CSF, monoclonal antibodies binding IL-6 directly—such as HZ-0408b [126]—via IT pseudodelivery might be an alternative route to target inflammation in NDD.

Lastly, a TREM2-activating antibody with a BBB transport vehicle enhances microglial metabolism in AD models [89] and tau pathology and neurodegeneration are associated with an increase in CSF sTREM2 [90]. However, some of these experiments can be interpreted as full-length TREM2 protecting rather than sTREM2 [26]. Therefore, while sTREM2 might be a suitable target via IT pseudodelivery in AD, more knowledge is needed to understand how, when, and in what cases this target might be of interest.

## 6. Conclusions

IT pseudodelivery of drugs is a novel concept to administer drugs to treat NDD, based on the CSF-sink therapeutic strategy by means of implanted devices. The mechanism of action relies on the properties of nanoporous membranes enabling selective molecular permeability.

Being an invasive procedure, the expected safety issues of IT pseudodelivery are related to device implantation and functioning. However, the promising advantages of IT pseudodelivery of drugs in terms of efficacy and drug-related safety would overcome the disadvantages.

Potentially, there are a number of NDD where IT pseudodelivery might be of interest. While there is a theoretical rationale supporting these indications, in vitro and in vivo testing is still lacking for most of them; therefore, the proposal should be considered just a therapeutic hypothesis today.

## Figures and Tables

**Table 1:** Summary of the potential molecular targets and the proposed classes of therapeutic agents to be administered via IT pseudodelivery for the most relevant neurodegenerative diseases.

Neurodegenerative Disease	Molecular Target	Proposed Classes of Therapeutic Agents
Alzheimer’s disease	Aβ	mAbs, aptamers [74,75,76,77,78,79,80]
		Enzymes [24]
		Albumin [81,82]
		Protein conformation stabilizers and aggregation inhibitors [81,82,83,84]
	Tau protein	mAbs, aptamers [85,86]
		Protein conformation stabilizers and aggregation inhibitors [87,88]
	sTREM2	mAbs, aptamers [89,90]
	IL-6	mAbs [91]
	TNF-α	fusion protein by recombinant DNA, mAb [92,93,94,95]
Parkinson’s disease and Dementia with Lewy bodies	α-synuclein	mAbs, aptamers [58,86,96,97]
		Enzymes [98]
		Protein conformation stabilizers and aggregation inhibitors [83,99]
	IL-6	mAbs [100]
	TNF-α	mAbs [101]
Multisystem Atrophy	α-synuclein	mAbs, aptamers [58,86,96,97]
		Protein conformation stabilizers and aggregation inhibitors [83,99]
		Enzymes [98]
Progressive supranuclear palsy	Tau	mAbs, aptamers [102,103]
		Protein conformation stabilizers and aggregation inhibitors [104]
Frontotemporal dementia	TDP43	mAbs, aptamers [105]
	Tau protein	mAbs, aptamers [85]
		Protein conformation stabilizers and aggregation inhibitors [104]
Amyotrophic lateral sclerosis	SOD	mAbs, aptamers [86]
		Protein conformation stabilizers and aggregation inhibitors [86,106,107]
	TDP43	mAbs, aptamers [108]
		Enzymes [109]
		Protein conformation stabilizers and aggregation inhibitors [110,111]
	Tau protein	mAbs, aptamers [85]
		Protein conformation stabilizers and aggregation inhibitors [104]
	IL-6	mAbs [112]
	TNF-α	mAbs [113,114]
Huntington’s disease and other diseases caused by polynucleotide-mutated repeats	mutant HTT protein and other polyQ-mutated proteins	mAbs, aptamers [115]
		Protein conformation stabilizers and aggregation inhibitors [116,117]

Abbreviations: mAbs—monoclonal antibodies, SOD—superoxide dismutase, sTREM2—soluble triggering receptor expressed on myeloid cells 2, Aβ—beta-amyloid, TNF-α—tumor necrosis factor α, IL-6—Interleukin 6.

## Data Availability

Not applicable.

## Supplementary Materials

The following supporting information can be downloaded at: https://www.mdpi.com/article/10.3390/pharmaceutics15030768/s1.
